# Oxidative stress mediates age-related hypertrophy of ligamentum flavum by inducing inflammation, fibrosis, and apoptosis through activating Akt and MAPK pathways

**DOI:** 10.18632/aging.104105

**Published:** 2020-11-20

**Authors:** Hao-Chun Chuang, Kun-Ling Tsai, Kuen-Jer Tsai, Ting-Yuan Tu, Yan-Jye Shyong, I-Ming Jou, Che-Chia Hsu, Shu-Shien Shih, Yuan-Fu Liu, Cheng-Li Lin

**Affiliations:** 1 Department of Orthopaedic Surgery, National Cheng Kung University Hospital, College of Medicine, National Cheng Kung University, Tainan, Taiwan; 2 Department of Physical Therapy, College of Medicine, National Cheng Kung University, Tainan, Taiwan; 3 Institute of Clinical Medicine, College of Medicine, National Cheng Kung University, Tainan, Taiwan; 4 Research Center of Clinical Medicine, National Cheng Kung University Hospital, College of Medicine, National Cheng Kung University, Tainan, Taiwan; 5 Department of Biomedical Engineering, National Cheng Kung University, Tainan, Taiwan; 6 Department of Clinical Pharmacy and Pharmaceutical Sciences, National Cheng Kung University, Tainan, Taiwan; 7 Department of Orthopedics, E-Da Hospital, I-Shou University, Kaohsiung, Taiwan; 8 Skeleton Materials and Bio-compatibility Core Lab, Research Center of Clinical Medicine, National Cheng Kung University Hospital, College of Medicine, National Cheng Kung University, Tainan, Taiwan; 9 Medical Device Innovation Center (MDIC), National Cheng Kung University, Tainan, Taiwan

**Keywords:** ligamentum flavum hypertrophy, oxidative stress, inflammation, fibrosis, apoptosis

## Abstract

The role of oxidative stress in ligamentum flavum (LF) hypertrophy has not been elucidated. We hypothesize that oxidative stress induces inflammatory responses and the subsequent fibrotic processes in LF, via activation of the Akt and MAPK pathways. Specimens of LFs were collected during surgeries for lumbar disc herniation (LDH) or lumbar spinal stenosis (LSS). Part of the LF specimens underwent analyses for ROS, fibrotic markers, and inflammatory mediators, with the remainder minced for cell cultures. The cell cultures were treated with H_2_O_2_, after which the cells were lysed and analyzed via western blotting. The specimens of the LSS patients showed increased infiltration of inflammatory cells and were stained positively for MMP-3, MMP-9, vimentin, and fibronectin. The LF of the LSS patients had increased oxidative stress and inflammation compared to that of the LDH patients. In vitro analyses demonstrated that oxidative stress rapidly activated the Akt and MAPK pathways. Inflammatory mediators, iNOS and NF-κB, and fibrotic markers, including TGF-β, β-catenin, α-SMA and vimentin, were significantly upregulated after induction of oxidative stress. Oxidative stress activated the intrinsic apoptotic pathway. These findings revealed that oxidative stress is one of the etiological factors of LF hypertrophy, which might provide new insights into treatment approaches.

## INTRODUCTION

Lumbar spinal stenosis (LSS) is a common cause of low back pain, sciatica and neurological claudication, accounting for 19.2% of cases over 60 years old [1, 2]. It is diagnosed when the diameter of the spinal canal or neural foramen is reduced. The majority of LSS involves the thickening of ligamentum flavum (LF), which covers the posterolateral surface of the spinal canal [3]. Hypertrophy of the LF is multi-causal, involving degenerative changes secondary to the aging process and mechanical stress [4–6]. Currently, management of symptoms resulting from LF hypertrophy depends primarily on oral analgesics and rehabilitation, with surgery as the last resort if other options fail [7].

In view of the lack of treatment options, physicians are breaking down the pathomechanisms of LF hypertrophy in search for a medically manageable etiology. Histological examinations of hypertrophic LF from LSS patients show that, compared to healthy LF tissue from lumbar disc herniation (LDH) patients, the bulk is composed of fibrotic tissue [8–10]. Increased expression of inflammatory-related genes was also demonstrated in hypertrophic LF tissue [9]. Inflammation in the LF was believed to promote fibrosis via a TGF-β1-mediated increase in the expression of collagen-1 and 3, and α-SMA [5, 8]. In a mouse model of LF thickening, macrophage infiltration was shown to be indispensable to the upregulation of collagen and vimentin production in fibroblasts [11]. On the other hand, subjecting cultured LF cells to cyclic tensile strain also resulted in TGF-β1-mediated upregulation of fibrotic markers, such as β-catenin, as well as collagen-1 and 3 [5, 12]. These findings indicated that fibrosis underlies the LF hypertrophy and results at least partially from inflammation and mechanical stress. However, other etiological factors may exist that induce inflammation in the absence of infection or autoimmunity.

Oxidative stress occurs when the generation of reactive oxygen species (ROS) overwhelms the capacity of antioxidant enzymes [13]. A diversity of signaling pathways are amenable to its redox modulation, including the mitogen-activated protein kinase (MAPK) pathways and Akt pathways [14–16]. Via the cross-talking of these pathways, ROS can alter the function of antiapoptotic proteins and activate the intrinsic pathway of apoptosis [16, 17]. It has been established that excessive production of ROS is involved in multiple aging disorders [18–20]. An abundance of ROS can induce strong inflammatory responses and give rise to the fibrosis of vital organs, including the heart, kidneys, lungs, and liver [18, 21–23]. In addition, repetitive mechanical stretching has also been reported to enhance the generation of ROS, to which the LF is continuously subjected during movements in the spine [12, 24, 25]. A recent study showed that the expression of catalase was reduced in hypertrophic LF tissue from LSS patients [26]. Together, this implies that oxidative stress may exist in hypertrophic LF tissue and activate the inflammation-fibrosis cascade. However, the existence and effect of oxidative stress in LF have not been elucidated in the literature, and the interplays between ROS and apoptosis in hypertrophic LF tissue have not been investigated.

In the current work, we aimed to determine whether oxidative stress plays a role in age-related LF hypertrophy and its potential molecular mechanism of action. We hypothesize that an excess of ROS induces inflammatory responses and results in fibrosis in LF cells. We also postulate that oxidative stress activates the MAPK and Akt pathways in LF, the cross-talking between which activates apoptotic pathways. To verify our hypothesis, we first evaluated the existence of oxidative stress and the expression of inflammatory and fibrotic markers in specimens of hypertrophic LF tissue. Next, we investigated the effect of oxidative stress on the signaling pathways, protein expressions, and apoptotic pathways via application of oxidative stress to primary cultures of LF cells

## RESULTS

### LSS patients were of older age and had more hypertrophic LF tissue compared to LDH patients

Of the 70 patients included in this study, the mean ages of the LSS group (49 patients) and the LDH group (21 patients) were 67 ± 9 and 37±10 years old, respectively, which means the age of the LSS group was significantly higher than that of the LDH group. (*p* < 0.001) The mean LF thickness was significantly higher in the LSS group compared to the LDH group, namely 5.06±1.15 mm and 2.71±0.54 mm, respectively (*p* < 0.001). The differences between the ratio of genders (*p* = 0.914) and BMI (*p* = 0.651) did not reach statistical significance. No statistical significance was notable between the two groups in respect of other comorbidities. (Table 1) These demographic data indicate that our population was a representative sample as they were compatible with the results of previous epidemiologic studies [2, 27].

**Table 1:** Patient demographics.

	**LDH**	**LSS**	***p* value^#^**
Number	21	49	
Gender (Male/Female)	8/13	18/31	0.914
Age (years)	37±10	67 ± 9	<0.001
BMI (kg/m^2^)	25.9±4.5	26.4±4.2	0.651
LF thickness (mm)	2.71±0.54	5.06±1.15	<0.001
DM*	4/21	16/49	0.248
Hypertension	4/21	19/49	0.107
Respiratory diseases^@^	2/21	5/49	0.931
Smoking	3/21	6/49	0.815

^#^ *p* value generated using Chi-square analysis or independent t-test; LDH, lumbar disc herniation; LSS, lumbar spinal stenosis; BMI, body mass index; DM, diabetes mellitus. ^@^ Respiratory diseases included chronic obstructive pulmonary disease, asthma, interstitial lung disease, etc.

### Up-regulation of fibrotic markers and infiltration of inflammatory cells in the specimens of hypertrophic LF from LSS patients

**Figure 1:**
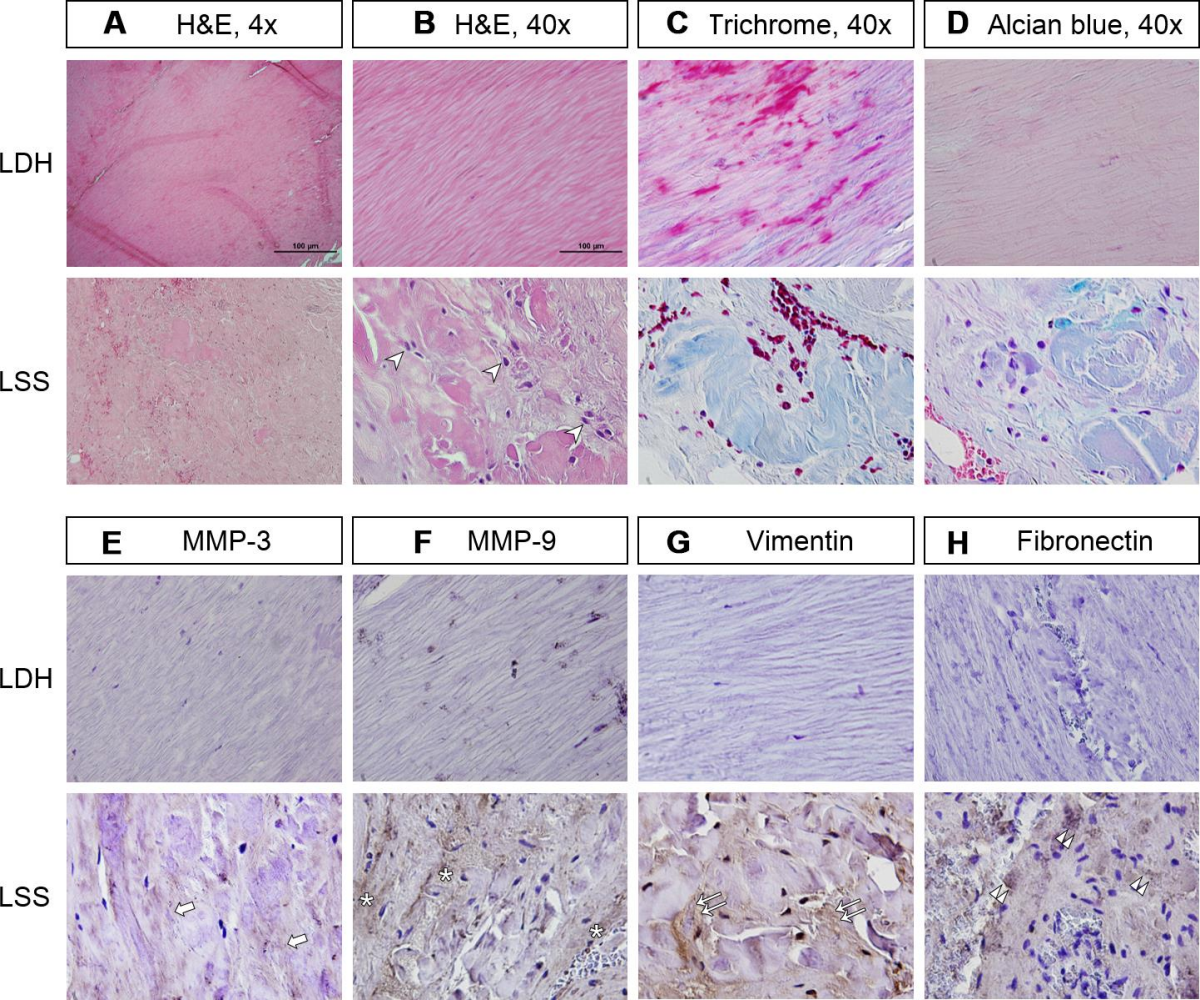
**The LF of lumbar spinal stenosis (LSS) patients was positively stained for inflammatory cell infiltration and fibrotic markers.** (**A**, **B**) In the LF of lumbar disc herniation (LDH) patients, the collagen fibers were well organized in a parallel order. In contrast, those from LSS patients showed signs of inflammation and fibrosis, including infiltration of inflammatory cells (arrowheads) and deposition of disorganized collagen fibers. Scale bar 100μm. (**C**) Masson’s trichrome staining revealed that the LF from LSS patients was stained a blue color with only traces of pink, further substantiating the increase in collagen deposition. (**D**) Alcian blue staining of the LF from LSS patients was stained a blue color, indicating increased deposition of glycosaminoglycans. (**E**, **F**) Immunohistochemical (IHC) analysis demonstrated that MMP-3 (arrows) and MMP-9 (asterisk) were positively stained on the LF specimens of LSS patients. (**G**, **H**) IHC analyses revealed that vimentin (double arrows) and fibronectin (double arrowheads) were positively stained on the LF specimens of LSS patients.

Compared to the LDH patients, the LF tissue from the LSS patients had increased deposition of disorganized collagen fibers and little residual elastin fibers, as shown by the H&E stains and Masson’s trichrome stain. The hypertrophic LF was also infiltrated with inflammatory cells. (Figure 1A–1D) Immunohistochemical analysis demonstrated that MMP-3 and MMP-9, which were involved in pro-fibrotic processes, were positively stained on the LF fibroblasts of the LSS patients. (Figure 1E, 1F) The expressions of vimentin and fibronectin were significantly enhanced in the LF cells from the LSS patients, further substantiating the occurrence of fibrosis. (Figure 1G, 1H) These results suggest that the inflammation-fibrosis cascade existed in hypertrophic LF.

### Increased oxidative stress in hypertrophic LF

**Figure 2:**
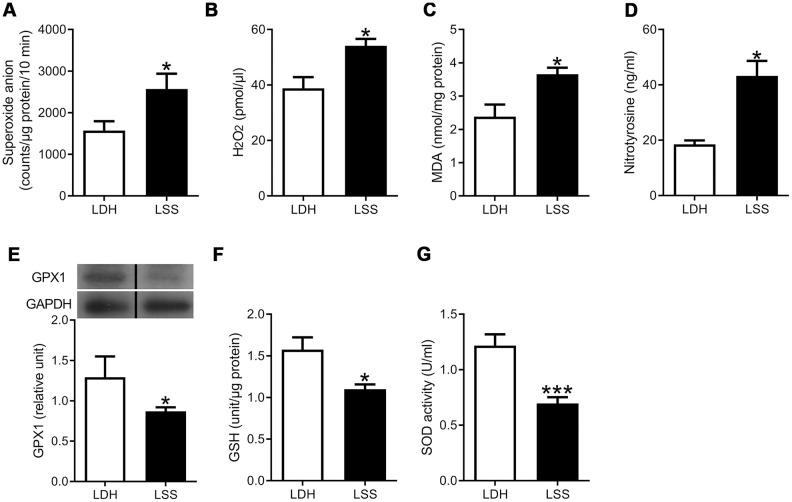
**Increased oxidative stress and decreased antioxidant defense were present in the hypertrophic ligamentum flavum.** The ROS levels, inclusive of (**A**) Superoxide anion and (**B**) Hydrogen peroxide, were higher in the LSS group compared to the LDH group. The expressions of (**C**) Malondialdehyde (MDA) and (**D**) Nitrotyrosine, which are markers of damage from oxidative stress, were higher in the LSS group. Meanwhile, the expressions of (**E**) Glutathione peroxidase (GPX1), (**F**) Glutathione (GSH), and (**G**) Superoxide dismutase (SOD) activity were decreased in the LSS group. (n = 21 and 43, respectively; * *p* < 0.05, *** *p* < 0.001 compared to the LDH group; the value was generated by unpaired t-test.) (The lanes on Figure 2E were run on the same gel but were noncontiguous.).

To compare the oxidative stress in hypertrophic and normal LF, we examined the levels of ROS radicals and markers of oxidative damage in the LF specimens obtained from the LSS and LDH patients. Significantly higher levels of superoxide anion (O2•-) and hydrogen peroxide (H_2_O_2_) were detected in the LSS group, compared to the LDH group (Figure 2A, 2B). In addition, the expression of malondialdehyde (MDA) and nitrotyrosine were elevated in the LF specimens of the LSS patients, indicating that lipid peroxidation and protein nitration occurred in hypertrophic LF tissue (Figure 2C, 2D).

The antioxidant capacities in healthy and hypertrophic LF tissues were also compared. The expression of glutathione peroxidase (GPx-1) was significantly reduced in the LSS group. In addition, the level of endogenous antioxidant, glutathione (GSH), and the antioxidant activity of superoxide dismutase (SOD) were significantly reduced in the LSS group as well (Figure 2E, 2F). These results imply that not only increased oxidation but also decreased reduction was present in the hypertrophy of LF. The severity of oxidative stress was shown to be associated with advanced age, obesity, and the degree of LF hypertrophy.

### Elevation of inflammatory mediators in hypertrophic LF tissue

**Figure 3:**
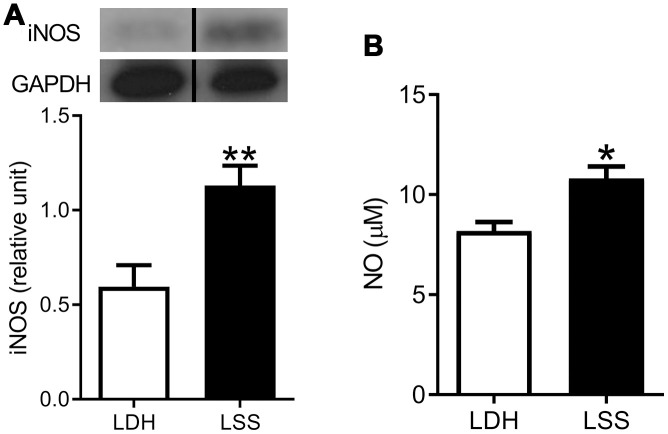
**Inflammation was present in the hypertrophic LF.** The expressions of (**A**) iNOS and (**B**) NO were increased in LF specimens from the LSS patients. (n = 21 and 43, respectively; * *p* < 0.05, ** *p* < 0.01 compared to the LDH group; the value was generated by unpaired t-test.) (The lanes on Figure 3A were run on the same gel but were noncontiguous.).

In addition to demonstrating the infiltration of inflammatory cells during pathological examination, we quantified the level of inflammatory mediators to corroborate the involvement of inflammation in LF hypertrophy. First, we demonstrated that the expression of inducible nitric oxide synthase (iNOS) was elevated in the homogenate of hypertrophic LF tissue. Next, the nitrite production assay showed that the nitrite level was increased, indicating that the level of NO was increased in hypertrophic LF (Figure 3). Together, these findings suggest that inflammation existed in concurrence with fibrosis in the hypertrophic LF tissue.

### Akt and MAPK signaling pathways were upregulated under oxidative stress in LF cells

**Figure 4:**
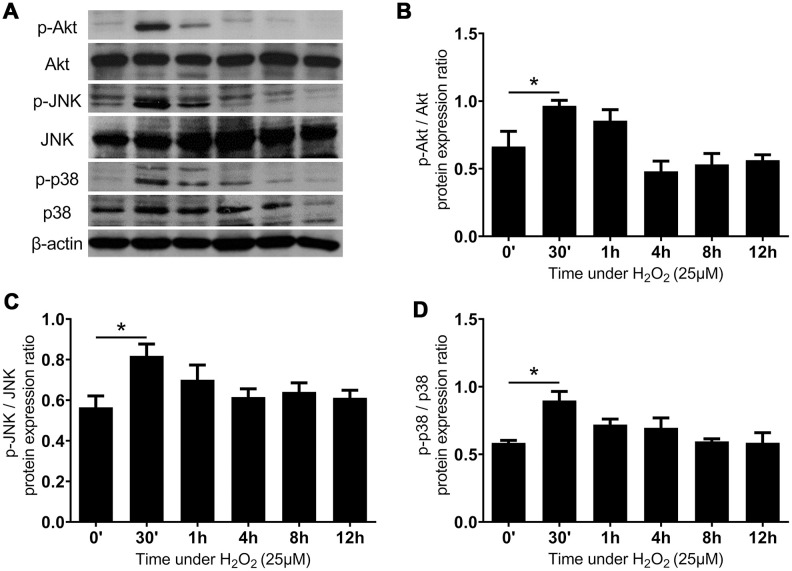
**The Akt, JNK, and p38 MAPK pathways were rapidly activated after administration of H_2_O_2_-induced oxidative stress.** (**A**) The expression of proteins of MAPK networks. The quantified protein expression ratio of (**B**) phosphorylated Akt (p-Akt) to total Akt, (**C**) phosphorylated JNK (p-JNK) to total JNK, and (**D**) phosphorylated p38 MAPK (p-p38) to total p38. The horizontal axis indicates the duration of subjecting the LF cells to the H_2_O_2_-induced oxidative stress. (n = 6; * *p* < 0.05 compared to the control group (0’); the value was generated by one-way ANOVA and post-hoc Tukey’s test.).

The Akt and MAP kinase families are known to mediate the adaptive responses to oxidative stress in the human body [28–30]. As expected, the oxidative stress induced by H_2_O_2_ enhanced the expression of phosphorylate Akt (p-Akt) in 30 minutes, the effect of which attenuated 1 hour after the addition of H_2_O_2_. We further examined the response of the MAPK pathways to the oxidative stress in LF cells. The protein expression of phosphor-JNK and phospho-p38 was significantly upregulated in 30 minutes, and then regressed to the non-stressed state after 1 hour. (Figure 4) These findings suggest that the LF cells employed the upregulation of the Akt, JNK, and p38 pathways to transduce signals and initiate adaptive responses under oxidative stress.

### Activation of inflammatory mediators and fibrotic markers in LF cells sustaining oxidative stress

**Figure 5:**
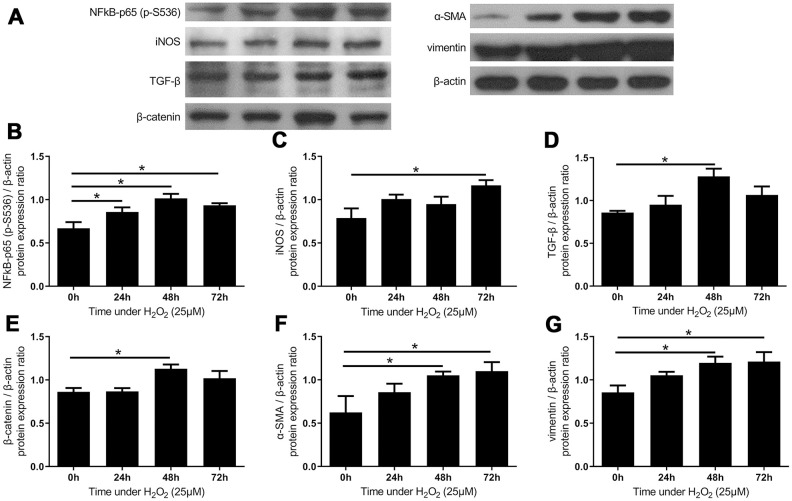
**The expressions of inflammatory markers and fibrotic markers increased after administration of H_2_O_2_-induced oxidative stress.** (**A**) The expression of inflammatory markers and fibrotic markers. The quantified protein expression ratio of (**B**) phosphorylated NF-κB p65 (S536) and (**C**) iNOS increased substantially under oxidative stress, suggesting the activation of inflammatory processes. The expressions of fibrotic signals such as (**D**) TGF-β and (**E**) β-catenin increased after 48 hours, followed by increases in fibrotic markers including (**F**) α-SMA and (**G**) vimentin. (n = 6; * *p* < 0.05 compared to the control group (0’); the value was generated by one-way ANOVA and post-hoc Tukey’s test.).

In the previous paragraphs, we mentioned that analyses of the specimens revealed that inflammation, fibrosis, and oxidative stress were present in the hypertrophic LF tissue. The oxidative stress was shown to effect rapidly on signaling pathways, so we further examined the influence of oxidative stress on the inflammatory and fibrotic markers in the LF cell cultures. The expression of phosphorylated NFκB-p65 (S536) elevated significantly after 24 hours, which results in enhanced transactivation. The expression of iNOS was also increased in the LF cell cultures treated with H_2_O_2_, as observed in the tissue homogenate of hypertrophic LF. (Figure 5B, 5C) These results lend support to the hypothesis that inflammation could be induced by oxidative stress.

**Figure 6:**
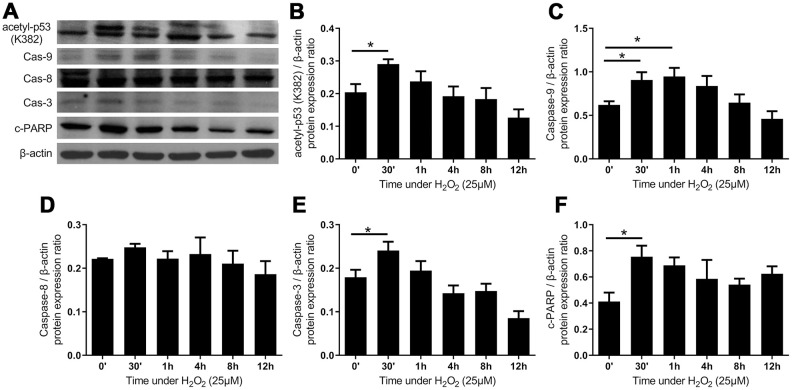
**The intrinsic apoptotic pathway was activated after administration of H2O2-induced oxidative stress.** (**A**) The expression of proteins of intrinsic apoptotic pathway. Quantified protein expression ratio of (**B**) acetylated p53 (K382) and (**C**) caspase-9 (Cas-9) were significantly increased after the addition of H2O2, indicating the activation of an intrinsic pathway. In contrast, the quantified protein expression ratio of (**D**) caspase-8 (Cas-8) did not vary significantly, implying that the extrinsic pathway was not involved. The increases in (**E**) caspases-3 (Cas-3) and (**F**) cleaved poly ADP-ribose polymerase (c-PARP) indicated the activation of apoptosis. (n = 6; * p < 0.05 compared to the control group (0’); the value was generated by one-way ANOVA and post-hoc Tukey’s test.).

The increases in the expression of TGF-β and β-catenin, both of which are essential mediators in fibrotic pathways, peaked at 48 hours and reached statistical significance. (Figure 5D, 5E) The expression of α-smooth muscle actin (α-SMA) and vimentin was upregulated in the H_2_O_2_-treated LF cell cultures as well. (Figure 6F, 5G) The expression of these fibrotic markers corresponded to the positive immunohistochemical stains on the hypertrophic LF-tissue specimens. The temporal sequence of upregulating signaling pathways and enhancement of protein expressions suggest that oxidative stress probably induced the inflammation-fibrosis cascade in the LF cells via activation of the Akt and MAPK pathways.

### Oxidative stress induced apoptosis via intrinsic pathway in LF cells

By functioning as redox messengers in the transduction of intracellular signals, ROS is of central importance in the regulation of apoptotic pathways [31]. First, we showed that the expression of acetylated p53 (K382) was significantly increased, which signified the activation of p53 to induce either growth arrest or cell death. Then, the expression of caspase 9, but not that of caspase 8, was up-regulated rapidly after treatment with H_2_O_2_. Downstream effector apoptotic proteins, including caspase 3 and cleaved poly (ADP-ribose) polymerase (c-PARP), significantly increased after 30 minutes of oxidative stress (Figure 6). Together, the upregulation of apical caspase in the intrinsic pathway but not in the extrinsic pathway denoted that oxidative stress presumably induced apoptosis of LF cells via the intrinsic pathway.

## DISCUSSION

Over the past decade, the impact of oxidative stress on vital organs has been studied extensively. ROS has been demonstrated to induce inflammatory and fibrotic processes in the heart, lungs, liver, and kidneys [21, 22, 32]. In this study, we showed that inflammation and fibrosis in hypertrophic LF tissue were associated with oxidative stress as well. First, hypertrophic LF specimens were stained positively for fibrotic markers and infiltration of inflammatory cells. Second, analyses of tissue homogenate revealed that inflammatory mediators were upregulated, ROS was increased, and the antioxidant defense was reduced. These findings corroborate the coexistence of inflammation, fibrosis, and oxidative stress in hypertrophic LF. In vitro studies subjecting LF cells to H_2_O_2_-induced oxidative stress showed that ROS rapidly activated the Akt, JNK, and p38 MAPK pathways. After triggering the signaling cascades, the oxidative stress enhanced the expression of inflammatory and fibrotic markers. In addition, the intrinsic apoptotic pathway was activated after the application of oxidative stress to the LF cells. Collectively, these findings suggest that oxidative stress contributed to the hypertrophy of LF via the triggering of inflammatory and fibrotic processes, as well as the apoptosis of LF cells. This knowledge could form the basis for new therapeutic approaches for lumbar spinal stenosis and enlighten us on how oxidative stress affects human LF tissue.

Fibrosis is known to lead to reduced organ function, but the progress of fibrosis cannot be curbed unless the cause is addressed [23]. For instance, renal fibrosis could occur after shockwave lithotripsy for nephrolithiasis, as the procedure injures renal parenchyma and induces inflammation and the production of ROS [33]. However, it was the revelation of elevated iNOS and NF-κB expressions that led to the introduction of curcumin and pyrolidium dithiocarbamate into treatment [34, 35]. On the other hand, the therapeutic mechanisms of pirfenidone and pioglitazone in tubulointerstitial fibrosis depended on their antioxidant properties, as they enhanced manganese SOD activity and diminished mitochondrial ROS by inhibiting the activity of cytochrome c oxidase [21, 36]. Past studies have mostly attributed LF fibrosis to age and chronic mechanical stress, which are irreversible factors [4–6]. Conventionally, these patients with LF hypertrophy were conservatively managed with oral analgesics or suggested surgical intervention [35, 37, 38]. However, oral nonsteroidal anti-inflammatory drugs are known to increase the risk of gastrointestinal and renal complications, while the cost and complications of surgical intervention can be excessive. In the current study, we showed that oxidative stress was present in the LF. Oxidative stress could induce fibrosis of the LF, the process of which also involved the upregulation of NF-κB and iNOS and the intrinsic mitochondrial pathway of apoptosis. These findings opened up possibilities for novel preventive or therapeutic approaches with anti-oxidant medication for the LF hypertrophy population.

The signal transduction pathways activated by oxidative stress and the interplays of these pathways with inflammatory and fibrotic responses in renal fibrosis have been studied extensively. In renal mesangial cells, intracellular ROS activates the PKC, MAPK, and JAK-STAT pathways to upregulate redox-sensitive transcription factors, including NF-κB, which further induces inflammation and excessive deposition of extracellular matrix proteins, thereby causing renal fibrosis [39]. Atorvastatin and melatonin have been reported to attenuate the detriments of nephrotoxins in mice models, where the mechanisms depended on inhibition of the p38 MAPK pathway and subsequent reduction in the expression of NF-kB and iNOS [40, 41]. The significance of the p38 MAPK pathway in tubulointerstitial fibrosis was further confirmed in a mice model, where inhibition of the p38 MAPK pathway reversed the increase in the expression of α-SMA, collagen I, and fibronectin after unilateral ureteral obstruction [42]. In tubular epithelial cells, the oxidative stress activates the JNK pathway and results in tubulointerstitial fibrosis via phosphorylating pro-fibrotic transcription factors, promoting TGF-β induced de-differentiation of tubular cells and directly inducing the intrinsic apoptotic pathway [43]. Little previous literature has addressed the intracellular signal transduction involved in the responses to oxidative stress in ligament cells. Repetitive mechanical stretching of parametrial ligament cells have been shown to induce oxidative stress and activate the PI3K/Akt signaling pathway [44, 45]. In the current study, we revealed that LF cells, which were also subjected to repetitive mechanical stretching in vivo, activated the Akt, JNK, and p38 MAPK pathways under oxidative stress. Considering the temporal sequence of rapid kinase activation and subsequent overexpression of inflammatory and fibrotic mediators, we speculated that the pathomechanism of LF fibrosis was similar to that of renal fibrosis. The ROS employed the MAPK pathway to promote the expression of inflammatory mediators, including iNOS and NO, and fibrotic mediators, including TGF-β and β-catenin. The fibrotic signals eventually enhance the translation of α-SMA and vimentin, leading to the advent of myofibroblasts and excessive deposition of extracellular matrix into LF tissue.

Apoptosis of cells in hypertrophic LF has been reported previously, the etiologies for which have been actively sought. Park et al. first reported that the expression of apoptotic markers was significantly higher in LSS patients than in those with LDH [3]. Cyclic stretching at 20% elongation was reported to enhance apoptosis in human LF cells, partially accounting for the increased cell deaths [25]. Enhanced apoptosis had a profound impact on the histological composition of ligamentum flavum. Nakama et al. revealed that normal fibroblasts were surrounded by thick collagen fibrils with the pericellular space filled; in contrast, apoptotic bodies were instead bordered by loose and thin fibrils [46]. Without healthy fibroblasts, the aberrant ECM composition and structure were not remodeled and conversely stimulated the remaining fibroblasts to further increase ECM production [47]. In consideration of the negative effects, researchers have been actively investigating how apoptosis is activated in fibrotic disorders. In the alveolar epithelial cells of idiopathic pulmonary fibrosis (IPF) patients, the ROS activates the ERK, JNK and p38 MAPK pathways and interacts with caspase 3 to upregulate cell death [48]. In the current study, the activation of intrinsic apoptotic pathways was also in close temporal proximity with that of the MAPK pathway. It was therefore deducted that activation of the intrinsic apoptotic pathway in the LF cells was driven by the cross-talking between the Akt and MAPK pathways and the interactions between the ROS and caspases.

There remain limitations to this study. The first was that the LDH cohort and the LSS cohort were not age-matched. The limitation resulted from the distinct epidemiological features of the two diseases: the LDH was most prevalent among patients in their 30s and 40s, while the lumbar spinal stenosis was most prevalent among those in their 60s [2, 27, 49]. However, the oxidative stress was inferred to be mediating the effect of age on LF rather than to be confounded by the difference in age, as pretreatment of reducing agents neutralized the fibrosis-inducing effect of H_2_O_2_ on LF cells (data not shown). Some previous studies on LFH went so far as to consider the LF from LDH patients as an ideal control group [11, 50–52]. The second was that multiple mechanisms were involved in the pathogenesis of LF hypertrophy, but oxidative stress was the only factor simulated in the current in vitro study. Other important mechanisms such as repetitive tensile stress sustained during flexion and extension of lumbar spine could be simulated in the future using 3D culture models of LF cells. In addition, we did not have an animal model to test the antioxidant medications mentioned in the previous paragraphs. By establishing an animal model of ligamentum flavum hypertrophy, we could investigate the effect of antioxidant and antifibrotic medications on the expression of inflammatory and fibrotic markers. Also, the influence of treatments on infiltrating inflammatory cells would be better elucidated by flow cytometry.

## CONCLUSIONS

**Figure 7:**
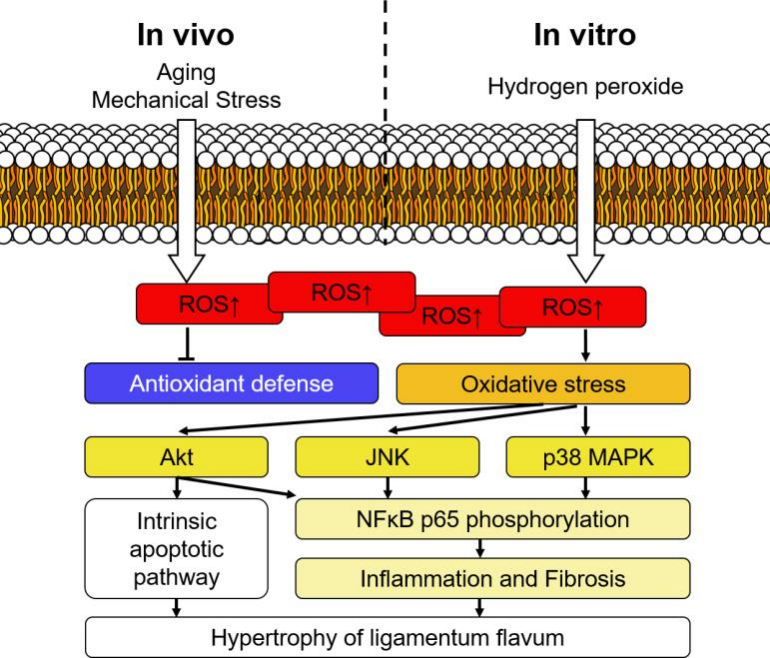
**Schematic diagram of signaling pathways involved in the hypertrophy of ligamentum flavum.** Intracellular ROS accumulated during the aging process and after repetitive mechanical stress in vivo, and the condition was simulated by hydrogen peroxide treatment in the current study. The upregulated oxidative stress subsequently activated Akt, JNK, and p38 MAPK pathways and triggered inflammation as well as fibrosis. Accumulation of fibrotic extracellular matrices eventually resulted in hypertrophy of the ligamentum flavum.

In conclusion, the present study demonstrated substantial evidence that oxidative stress contributes to the hypertrophy of LF. The ROS activates the Akt, JNK, and p38 MAPK pathways and promotes the expression of inflammatory mediators as well as fibrotic markers. The intrinsic apoptotic pathway was also activated following exposure to the oxidative stress. (Figure 7) These findings revealed that oxidative stress is one of the etiological factors of LF hypertrophy, and therefore a possible pharmacological target.

## MATERIALS AND METHODS

### Reagents and antibodies

Griess reagent, 5,5’-Dithiobis (2-nitrobenzoic acid) and Lucigenin were purchased from Sigma-Aldrich. Hydrogen peroxide 33% was procured from PanReac AppliChem. The following antibodies were used in this work: Anti-fibronectin was obtained from GeneTex. Anti-MMP-9 and anti-Bcl2 were obtained from Proteintech. Anti-vimentin, anti-MMP-3, Anti-Akt, Anti-p38, Anti-Cleaved PARP1, Anti-p53 (acetyl K382), Anti-JNK, Anti-alpha smooth muscle Actin, Anti-NF-kB p65 (phospho S536), and anti-β catenin were all obtained from Abcam (Cambridge, UK). Anti-cleaved caspase-3, anti-cleaved aspase-9, and anti-cleaved caspase-8 were obtained from Cell Signaling. Rabbit Polyclonal JNK1/2/3 was obtained from ORIGENE. Anti-beta Actin was obtained from Thermo Fisher Scientific. Peroxidase AffiniPure Goat Anti-Rabbit IgG and Peroxidase AffiniPure Goat Anti-Mouse IgG were obtained from Jackson ImmunoResearch Inc. The following ELISA kits were used in this work: nitrotyrosine ELISA kit (Abcam, ab113848), Hydrogen Peroxide Colorimetric/Fluorometric assay kit (Biovision, K265-200), malondialdehyde assay kits (Northwest Life Sciences Specialties, NWK-MDA01) and Superoxide Dismutase assay kit (Cayman chemical, 706002).

### Participants and MRI analysis

**Figure 8:**
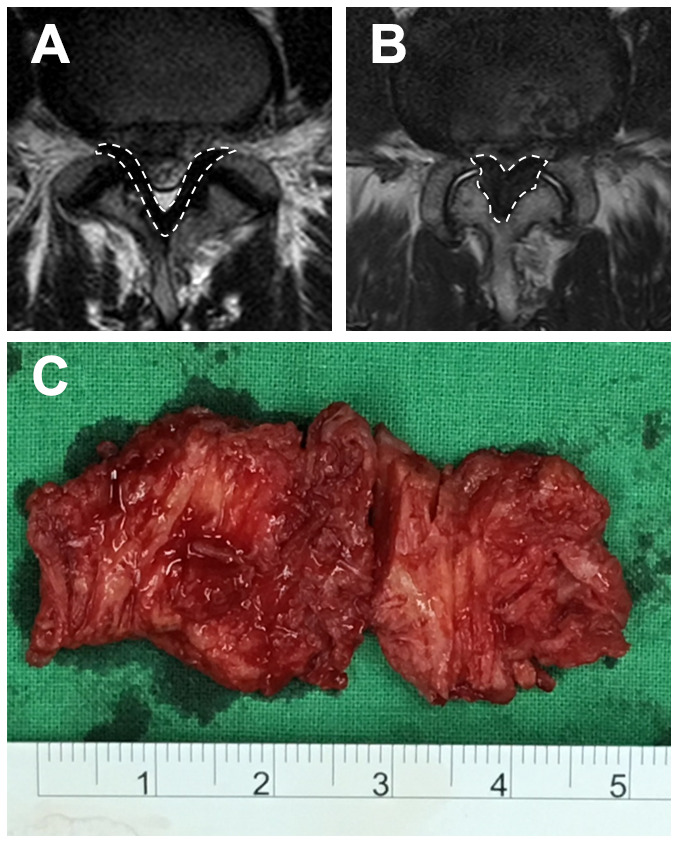
**MRI image and the LF specimen.** The T2-weighted image of the LF, surrounded by broken lines, at the L4/5 level of (**A**) an LDH patient and (**B**) an LSS patient. (**C**) A hypertrophic LF specimen.

A total of 70 patients undergoing surgery at a tertiary referral hospital in southern Taiwan were included. Patients with a previous history of epidural or selective nerve-root blocks, malignancy, vertebral fracture, vertebral osteomyelitis or previous spine surgery were excluded. All patients underwent imaging with magnetic resonance images (MRI) preoperatively. The maximum thickness of the LF was measured using the axial T2-weighted image at the facet-joint level of the lesion [53]. (Figures 8A, 8B) The radiographic analyses were independently performed by two senior spine surgeons who were not involved in the care of the patients. Each observer independently measured the thickness of the LF twice, with the average of the four measurements (two from both surgeons) used as the final thickness.

Specimens of hypertrophic LF were collected aseptically from 49 patients affected with lumbar spinal stenosis (LSS group) during posterior lumbar decompression surgery. Meanwhile, samples of healthy LF tissue were collected from 21 patients affected with lumbar disc herniation (LDH group) during lumbar discectomy. The LF at the diseased lumbar level was harvested en bloc (Figure 8C), and the epidural fat and the bone-ligament junction were accurately removed. Six specimens from the LSS group were used for isolation of LF cells. Halves of the remaining 64 LF specimens were fixed in 4% neutral formalin, decalcified with 20% EDTA, and embedded in paraffin for subsequent histologic analyses. Meanwhile, the other halves were kept in a freezer at -80° C for subsequent assays of oxidative stress and antioxidant capacity.

### Human LF cell isolation

Cells were isolated from the 6 LF specimens, as described by previous studies [54, 55]. The surgical LF specimens were first washed with phosphate-buffered saline (PBS) several times, after which any residual adipose or connective tissues still attached to the specimen were carefully removed. The LF specimens were then minced into pieces of approximately 0.5mm^3^ and placed in a 10 cm culture dish with 10 ml of high glucose Dulbecco’s Modified Eagle’s medium (DMEM; Gibco BRL, Melbourne, Australia), with 10% fetal bovine serum (FBS; Gibco BRL) and 100U/ml penicillin. The specimens were incubated at 37° C in an air-humidified incubator containing 5% CO_2_. The culture medium was changed twice weekly. When the cells reached confluence in the dishes, they were treated with 0.25% trypsin and subcultured. Cells after the third passage were used for experiments. The LF cells were treated with 25 μM H_2_O_2_ to induce oxidative stress, as described by Upadhyay et al [56].

### Histologic analyses

### Special stains

Two consecutive sections (4-μm thick) were cut on a microtome and stained by hematoxylin-eosin (H&E) and Masson’s trichrome stains, respectively. H&E stain was used to evaluate the elastin degradation (loss, fragmentation and disorganization) while Masson’s trichrome stain was used to determine the degree of fibrosis. Histologic analyses were independently performed by two investigators on 10 randomly selected, high-power fields (× 40) of each sample.

### Immunohistochemistry

LF tissue samples were fixed in 4% paraformaldehyde (PFA), embedded in paraffin, and then sectioned. After the sections were pretreated with Target Retrieval Solution, pH 9 (Tris/EDTA buffer, pH 9), the endogenous peroxidases were blocked using periodic acid. IHC stains were then performed as previously described. The antibodies against fibronectin (GTX61206, 1:250, GeneTex), MMP-9 (10375-2-AP, 1:250, Proteintech), and vimentin (550513, 1:50, BD Biosciences) were used. For negative controls, normal mouse IgG (Dako, Copenhagen, Denmark) and normal rabbit IgG (Dako) were used as a primary antibody. After the sections were washed, they were incubated with primary antibody for 1 h. The protein was visualized by incubating the sections with diaminobenzidine (DAB). All slides were stained in one session.

### Analyses of oxidative stress and antioxidant capacity

### Superoxide anion levels

Superoxide anion levels were measured with a high-performance chemiluminescence (CL) analyzer (model CLA-2100; Tohoku Electronic Industrial Co. Ltd., Rifu, Japan). Before the experiment, the cooler was pre-cooled to 5° C. Four hundred μl of nerve homogenate was mixed with 200 μl of PBS in a stainless dish, after which the background CL count was read for 60 sec. One hundred μl of lucigenin (17 mM dissolved in PBS, for determination of superoxide anion, hydroxyl radical, and peroxy-nitrite, respectively) was injected into the CL analyzer and the CL was counted for another 20 minutes at 10-sec intervals. The data were then analyzed by Chemiluminescence Analyzer Data Acquisition Software (CLA-DAS) (Tohoku Electronic Industrial Co.).

### Lipid peroxidation measurements

Malondialdehyde (MDA) is generated by exposure to ROS and free radicals and therefore a biomarker of lipid peroxidation. LF homogenate (200 μl) was taken for the MDA measurement by using a commercial assay kit (Lipid Peroxidase Assay Kit; Calbiochem-Novabiochem GmbH [now Merck Biosciences GmbH], Darmstadt, Germany). The LF sample (200 μl) was mixed with 650 μl of assay solution and 150 μl of 12N HCl. After incubation at 45° C for 60 min, thee reaction was stopped by immerging the sample tube into ice-cold water for 1 min. Fluorescence intensity was then assessed at 586 nm using a spectrophotometer (DU 640B; Beckman, Fullerton, CA).

### Nitrotyrosine, hydrogen peroxide, GSH and SOD activity assay

The tissue homogenates were re-suspended in 4 volumes of 6% metaphosphoric acid at 4° C, mixed thoroughly, and centrifuged at 3000G for 10 minutes at 4° C. One hundred microliters of supernatant was used to assess the nitrotyrosine (Nitrotyrosine Assay Kit; Abcam ab113848), hydrogen peroxide (hydrogen peroxide Assay Kit; Biovision catalog #K265-200), GSH (Glutathione Assay Kit; Calbiochem-Novabiochem) and SOD (Superoxide dismutase Assay Kit; Cayman item NO. 706002) levels with the respective assay kits, and the spectrophotometer was read at 400 nm.

### Western blot analysis

Tissue was homogenized in ice-cold lysis buffer (1:10; w/v) containing 20 mM of HEPES (pH 7.2), 1% Triton X-100, 10% glycerol, 1 mM of PMSF, 10 μg/ml of leupeptin, and 10 μg/ml of aprotinin. We centrifuged this solution at 12,000 rpm for 30 min and determined the protein concentration in the supernatant using protein assay dye (Bio-Rad Laboratories, Hercules, CA) with bovine serum albumin (BSA) as the standard. We loaded 50 μg of protein on 8% and 10% sodium dodecyl sulfate polyacrylamide gel for electrophoresis and then transferred it to nitrocellulose sheets (NEN Life Science Products, Inc, Boston, MA) in a transfer apparatus (Bio-Rad) running at 1.2 A for 2 h. After blocking the blots in 5% nonfat skim milk in TBST, we incubated the blots with primary antibodies, which were against target protein [GPX-1,iNOS], in 5% nonfat skim milk and then again with anti-rabbit IgG conjugated with alkaline phosphatase (dilution 1:5000; Jackson Immuno Research Laboratories, Inc, Philadelphia, PA). Immunoblots were developed using BCIP/NBT solution (Kirkegaard and Perry Laboratories, Inc, Baltimore, MD). The proteins were quantified using densitometry with the ImageJ computer program (National Institutes of Health; available at http://rsb.info.nih.gov/ij/).

### Nitrite production measurements

The amounts of nitrite, an indicator for nitric oxide production, were measured after the Griess reaction by incubating 100 mL of tissue lysate with 100 mL of Griess solution at room temperature for 20 min. The absorbency was measured at 550 nm using a spectrophotometer. Nitrite concentration was then calculated by comparing it with a standard solution of known sodium nitrite concentrations.

### Statistics

Data were presented as means ± standard deviation (SD). The student t-test was used to compare the differences between the LSS and LDH groups. One-way ANOVA, followed by the post-hoc Tukey’s test, was used to compare the data of cell cultures harvested at different time points. The association between LF thickness and age was examined using Pearson’s correlation coefficient; meanwhile, the association between gender and diagnosis was examined using Chi-square analysis. All data in this study were analyzed using SPSS version 17 (SPSS Inc., Chicago, IL, USA).

### Study approval

The study protocol was approved by the institutional review board of National Cheng Kung University Hospital (A-ER-104-348), and each participant provided written informed consent.
